# The prognostic importance of features of myometrial invasion in endometrial endometrioid carcinoma

**DOI:** 10.1007/s00404-025-08103-6

**Published:** 2025-07-16

**Authors:** Maya Pasternak, Roy Kessous, Benzion Samueli, Jacob Dreiher, Mihai Meirovitz, Sharon Davidesko, Ruthy Shaco Levy

**Affiliations:** 1 Faculty of Health Sciences, Ben Gurion University of the Negev, Beer Sheva, Israel; 2 Department of Obstetrics and Gynecology, Soroka University Medical Center, Beersheba, Israel; 3 The Institute of Pathology, Soroka University Medical Center, Beersheba, Israel; 4 The Institute of Pathology, Barzilai University Medical Center, Ha’Histadrut Street 2, Ashkelon, Israel

**Keywords:** Endometrial carcinoma (EC), Myometrial invasion (MI), Tumor-free distance (TFD), International Federation of Gynecology and Obstetrics (FIGO)

## Abstract

**Purpose:**

The depth of myometrial invasion (MI) is known to have a prognostic value in endometrial carcinoma (EC), and the FIGO 50% cutoff is widely accepted; however, recent studies have suggested other measurements such as the absolute depth of invasion and tumor-free distance (TFD) from the serosal surface to also be predictive. The aim of this study was to assess the association between the FIGO cutoff and other measures with overall survival and disease-free survival of patients.

**Methods:**

This is a retrospective analysis of a cohort of 248 women diagnosed with stage I endometrioid endometrial carcinoma, treated at Soroka University Medical Center between 2006 and 2020. Clinical and pathological data were collected and analyzed. ROC analysis was used to define the best cutoffs in all three categories (MI, absolute depth and TDF). Survival analyses were then conducted using Kaplan–Meier curves, log-rank tests, and Cox proportional hazards regression.

**Results:**

Absolute myometrial invasion (MI) to the depth of 1 cm significantly predicted overall survival (log-rank, *p* = 0.009) in univariate analysis; however, this significance was not maintained in multivariate analysis. Additionally, a 33% MI cutoff demonstrated potential for better outcome prediction as compared to the commonly used 50% MI threshold, though it did not reach statistical significance. Tumor-free distance (TFD) from the serosal surface was not significantly associated with outcome.

**Conclusions:**

MI depth of more than 1 cm may serve as a meaningful prognostic indicator. Additionally, a cutoff of 33% MI probably has a better prognostic value than the current 50% cutoff.

These findings show a promising direction for future research, emphasizing the need for larger cohorts and multicenter studies to confirm our findings.

## Introduction

Cancer of the uterine corpus is the most common gynecological cancer in the developed world [1] with a rise in incidence and mortality in recent years [2]. Endometrial carcinoma (EC) is the most common type of uterine cancer and accounts for 75–80% of all uterine malignancies [3]. EC is historically classified into two groups, type I and type II, based on epidemiology, histopathology, prognosis, and treatment [3]. Type I EC accounts for most cases (85%) with tumors of endometrioid histology. Type I EC have relatively good five-year survival and low recurrence rate. They primarily arise in obese perimenopausal women with a background of endometrial hyperplasia due to increased exposure to estrogen [4].

The prognosis, as well as management decisions, in patients diagnosed with EC are based on the International Federation of Gynecology and Obstetrics (FIGO) staging system using, among other features, evaluation of tumor architectural components and nuclear grading [5, 6]. The 2023 FIGO revision [6] represents a significant shift in endometrial cancer staging by integrating both histological type and grade as key components of the staging criteria. The new system distinguishes between less-aggressive histological types (comprising low-grade endometrioid carcinomas) and aggressive types, with some low-grade endometrioid tumors being down-staged based on their favorable prognostic features. However, the implementation of the 2023 FIGO system is still in transition, and this ongoing change period highlights the importance of careful evaluation of prognostic factors including myometrial invasion.

One very important prognostic factor in the FIGO staging system is the extent of tumor myometrial invasion (MI). The extent of tumor MI is typically calculated as the percentage of tumor invasion of the myometrium layer out of the total myometrial wall thickness [7]. MI holds great importance as a prognostic index and therefore is an integral part of the pathological evaluation completed for every sample suspected of endometrial cancer according to the protocol of the College of American Pathologists (CAP) [8]. Patients with deep MI may require a more advanced surgical staging procedure, as well as possible consideration of adjuvant treatment, due to the higher risk of pelvic lymph node metastasis and poorer prognosis [9]. In addition, deep MI correlates with a higher incidence of other poor prognostic factors such as uterine cervix extension, positive peritoneal fluid cytology, adnexal involvement, and lymph-vascular space invasion [7].

Historically, the FIGO staging system classified deep myometrial invasion using a one-third (33%) threshold before transitioning to a one-half (50%) cutoff in the 2009 update [10]. Studies at the time suggested that 50% invasion correlated more strongly with lymphovascular space invasion (LVSI) and nodal metastases [11, 13]. The adoption of a 50% threshold aimed to enhance the prognostic accuracy of the staging system and has proven to be a relatively reliable predictor of outcome [11–13]. The change was also made to improve reproducibility among pathologists, as differentiating between one-third and one-half invasion was often challenging. However, whether this change truly optimized prognostic accuracy remains a topic of debate, and the specific rationale for choosing 50% is not clear [14, 15].

Pathologically, invasion of the myometrial wall is sometimes challenging to assess, since the endometrial-myometrial border is not always sharp and clear. Consequently, pathologists occasionally use estimations to conclude whether the invasion of the myometrial wall is greater than or less than 50 percent [14, 16]. These estimations include using the arcuate vascular plexus (AVP) in the myometrium as a landmark for over fifty percent invasion and assuming that the anterior and posterior uterine walls are of the same thickness [16].

Given those challenges, previous studies evaluated the tumor-free distance from the uterine serosa (TFD) as a possible, more accurate, and objective assessment of myometrial invasion [17–19]. In addition, recent studies have tried to evaluate whether assessing the overall depth of invasion might be the best variable to correlate with outcome [20]. Part of these studies showed that TFD to the serosa, as well as absolute depth of invasion (DOI) [18], may be better prognostic indicators compared to the percentage of myometrial invasion [17, 20, 21].

The aim of the current study, focusing on a population of patients diagnosed with early-stage EC, is to assess and compare the prognostic value of various depth of invasion measurements. We first aim to understand whether the current FIGO classification cutoff of 50% is a reliable predictor of outcome. In addition, we compared this cutoff to the absolute DOI, as well as TFD, in order to assess whether they offer additional important information or even better prediction. Given the complexity of management decisions in early-stage EC, applying more accurate methods for identifying patients at higher risk for disease recurrence and mortality would facilitate adequate treatment, ultimately resulting in improved prognosis.

## Methods

This is a retrospective cohort study based on a population of women diagnosed with early-stage endometrioid type endometrial carcinoma, treated at Soroka University Medical Center between 2006 and 2020. All patients underwent surgical staging as part of their treatment. Participants were included if they were 18 or older, had a confirmed diagnosis of Stage IA or IB endometrioid carcinoma. Furthermore, all patients had complete clinical follow-up data available for at least five years post-surgery, allowing for long-term survival analysis. Patients were excluded from the study if they had been diagnosed with a non-endometrioid type endometrial malignancy, or had advanced disease stage at diagnosis. Additionally, cases with incomplete surgical staging procedure or missing follow-up data were excluded from the analysis to ensure the robustness of the study findings.

In addition to the primary clinical data, the severity of comorbid diseases was recorded and scored using the Charlson Comorbidity Index (CCI) [22], accounting for both the number and severity of the patient’s comorbid conditions. CCI scores were used to classify patients into five categories based on their overall comorbidity burden: no comorbidity (CCI score of 0), low comorbidity (CCI score of 1), moderate comorbidity (CCI score of 2), high comorbidity (CCI scores of 3–4), and severe comorbidity (CCI score ≥ 5). This enabled a comprehensive assessment of how comorbidities might influence patients survival and clinical outcomes in the context of endometrial carcinoma.

Data was collected from hospital database which provided comprehensive clinical and pathological information. Pathological data were extracted from histopathology reports and included tumor grade, depth of myometrial invasion (both as a percentage and in millimeters), and the TFD. For the purpose of this study, we relied on the original pathology reports at the time of hysterectomy for data extraction, and reviewed the slides on an as-needed bases (eg missing or unclear data points). Clinical outcomes, including overall and disease-free survival were analyzed.

### Data Analysis

Descriptive statistics were used to summarize the baseline characteristics of the study population. Categorical variables were expressed as frequencies and percentages, while continuous variables, such as patient’s age and depth of invasion, were summarized using means, standard deviations, medians and interquartile ranges. To explore associations between depth of myometrial invasion and characteristics of the study population, Chi-square tests were performed. To evaluate the prognostic value of the different invasion parameters, ROC (Receiver Operating Characteristic) curve analysis was performed for each variable, with the area under the curve (AUC) used to determine the most accurate predictor of survival and various cut-offs checked for sensitivity and specificity. Kaplan–Meier survival curves were constructed to estimate overall survival and disease-free survival. Log-rank test was used to compare survival between the different invasion parameter groups. Univariate Cox proportional hazards regression was applied to assess the relationship between each variable and survival outcomes. Variables with a p-value of less than 0.05 in the univariate analysis were entered into a multivariate Cox regression model to adjust for potential confounders, including age and tumor grade. Assuming a 20% difference in survival between patients with more invasive cancer vs. less invasive cancer and a significance level of 0.05 yields a sample size of at least 180 patients to ensure a power of 90% (computed using sample size immediate command in Stata version 12).

## Results

A total of 248 women with stage I endometrioid endometrial carcinoma were included in the study. The mean age of the cohort was 64 years (SD ± 8.5). Tumor characteristics and patient outcomes varied considerably within the cohort, as summarized in Table 1. Regarding tumor grade, the majority of cases were FIGO grade 1 (55.2%, n = 137), followed by grade 2 (29.8%, n = 74), and grade 3 (14.9%, n = 37). During the follow-up period, 31.9% of patients (n = 79) had died, with a mean survival time of 68.3 months (SD ± 52.9). Disease relapse occurred in 28.6% (n = 71) of patients.

**Table 1:** Baseline characteristics of the study population

	(N = 248)
**Age (years)**	
Mean (SD)	64.2 (± 8.5)
median [min, max]	66.9 [41.5, 84.8]
**Death (n = 247)**	79 (32.0%)
**Survival time (months)**	
Mean (SD)	68.3 (± 52.9)
Median [min, max]	51.3 [0.5, 185.1]
**Relapse**	71 (28.63%)
**Smoking**	32 (12.9%)
**FIGO grade**	
1	137 (55.2%)
2	74 (29.8%)
3	37 (14.9%)
**Charlson comorbidity index**	
No comorbidity	30 (12.1%)
Low comorbidity	33 (13.3%)
Moderate comorbidity	45 (18.1%)
High comorbidity	48 (19.4%)
Severe comorbidity	92 (37.1%)
**Comorbid conditions**	
Congestive heart failure	8 (3.2%)
Peripheral vascular disease	6 (2.4%)
Diabetes melitus	58 (23.4%)
Chronic kidney disease	10 (4.0%)
Myocardial infarction	10 (4.0%)
Chronic obstructive pulmonary disease	18 (7.3%)
Liver disease	20 (8.1%)

Comorbidities were prevalent in our study population, as assessed by the Charlson Comorbidity Index. A significant proportion of patients (37.1%, n = 92) had severe comorbidity, while only 12.1% (n = 30) had no comorbidity. The remaining patients were distributed across low (13.3%, n = 33), moderate (18.1%, n = 45), and high (19.4%, n = 48) comorbidity categories. The most common comorbidity was diabetes mellitus, present in 58 participants (23.4%). Chronic obstructive pulmonary disease (COPD) was observed in 18 participants (7.3%), while liver disease was noted in 20 participants (8.1%).

Table 2 presents the characteristics of myometrial invasion in our cohort. The mean depth of myometrial invasion was 0.68 cm (SD ± 0.62), with a median of 0.5 cm. When expressed as a percentage, the mean myometrial invasion was 37% (SD ± 30%), with a median of 31%. The mean distance from the serosal surface was 1.21 cm (SD ± 0.78), with a median of 1.1 cm (range: 0–6.5 cm). These data demonstrate considerable variability in the extent of myometrial invasion among patients with early-stage endometrial cancer.

**Table 2:** Myometrial invasion characteristics

	**(N = 248)**
**Depth of myometrial invasion (Cm)**	
Mean (SD)	0.68 (± 0.62)
Median [min, max]	0.5 [0, 3.9]
**Depth of myometrial invasion (%)**	
Mean (SD)	0.37 (± 0.30)
Median [min, max]	0.31 [0, 1]
**Distance from serosal surface (Cm)**	
Mean (SD)	1.21 (± 0.78)
Median [min, max]	1.1 [0, 6.5]
**Lymphovascular invasion**	41 (16.5%)

**Fig. 1:**
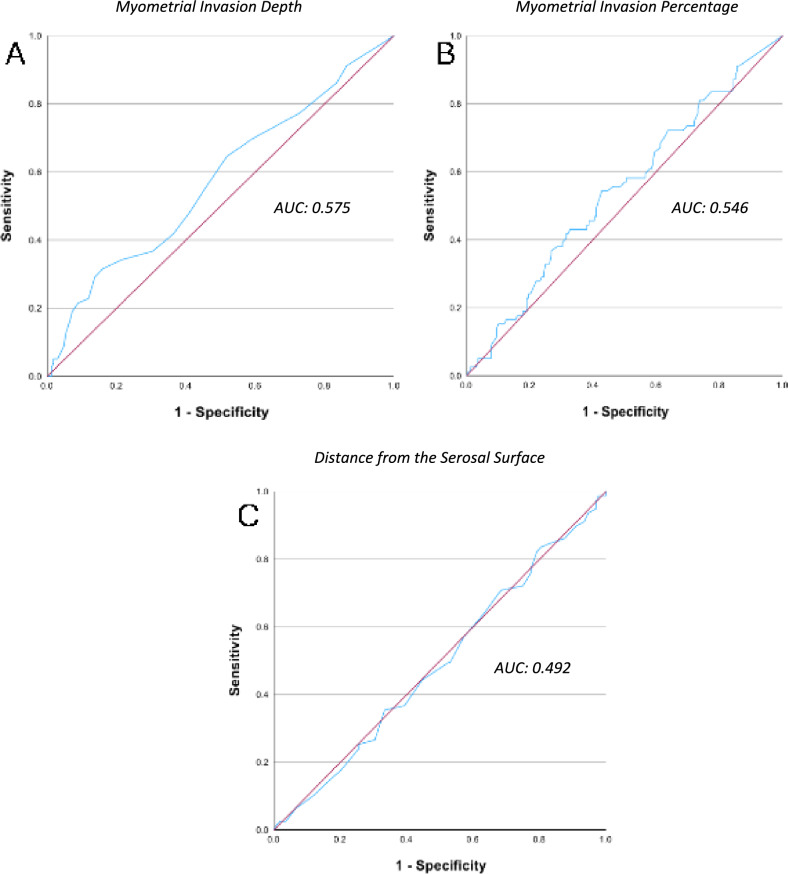
ROC curves for myometrial invasion depth, percentage, and distance from serosa. The red diagonal line represents the performance of a random classifier. The blue lines represent the discriminatory performance of the various prognostic factors

To evaluate and compare the predictive ability of the myometrial invasion depth, percentage, and distance from the serosal surface, we performed ROC curve analyses for all three estimators (Fig. 1). The area under the curve (AUC) for the depth of myometrial invasion (in cm) was 0.575 (95% CI 0.497–0.653; p = 0.05), indicating a modest discriminatory ability (Fig. 1A). The AUC for the percentage of myometrial invasion was 0.546 (p = 0.244), with optimal cutoff of 33%, yielding the highest correlation with outcome (Figs. 1B). As for the distance from the serosal surface, AUC was 0.492 (p = 0.839) (Fig. 1C).

**Fig. 2:**
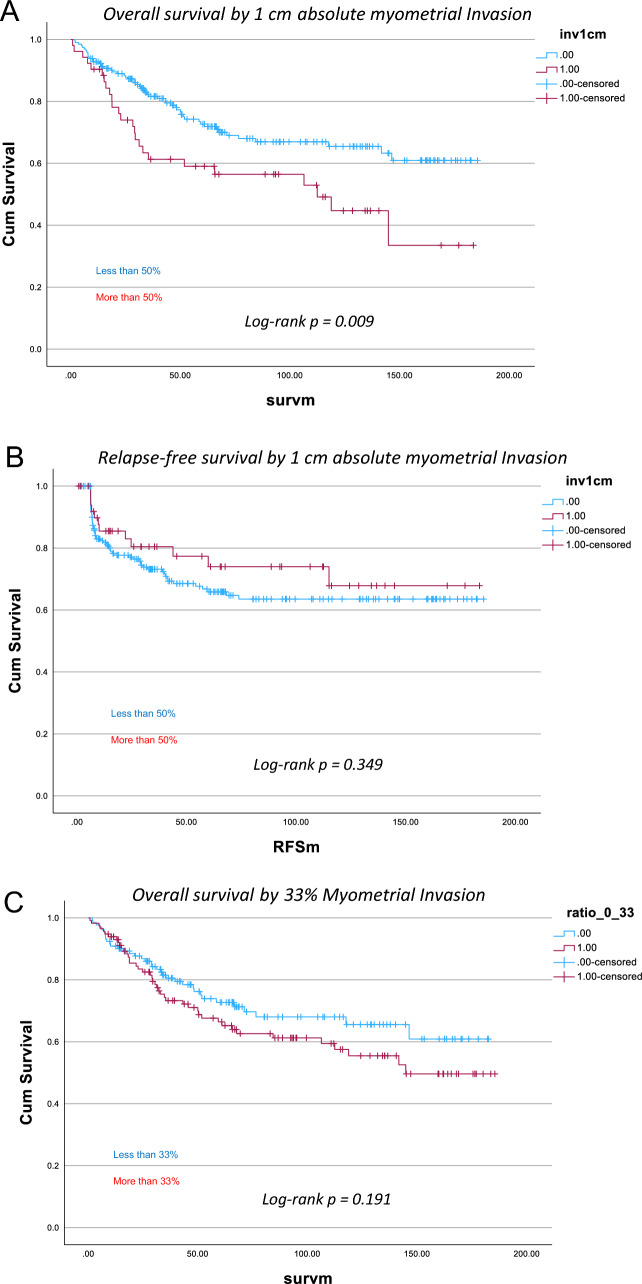

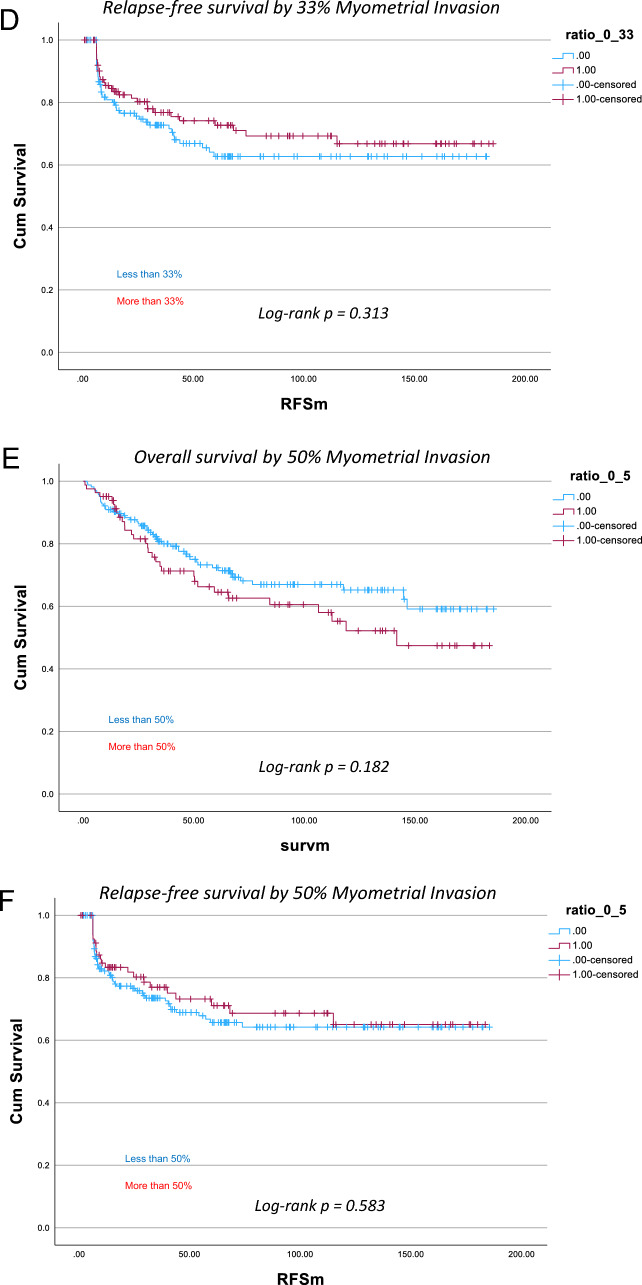
Kaplan–Meier survival curves comparing 1 cm, 33% and 50% myometrial invasion for both overall survival and relapse-free survival

For mortality, the log-rank test comparing survival distributions at the 50% myometrial invasion threshold was not statistically significant (p = 0.182; Fig. 2E). However, when using the 1 cm invasion cutoff, the log-rank test showed a significant difference in survival distributions (p = 0.009; Fig. 2A), suggesting that the 1 cm threshold may be a more relevant predictor of overall survival. The 33% invasion cutoff also demonstrated a trend toward improved survival prediction, though it did not reach statistical significance (*p* = 0.191; Fig. 2C).

Similar comparisons were conducted for relapse-free survival; however, none of the assessed myometrial invasion parameters demonstrated statistically significant associations. The 50% threshold (p = 0.583; Fig. 2F), the 1 cm absolute invasion cutoff (p = 0.349; Fig. 2B), and the 33% cutoff (p = 0.313; Fig. 2D) all failed to show significant differences in relapse-free survival.

We proceeded with Cox proportional hazards regression analysis to further assess the impact of myometrial invasion depth (1 cm) on overall survival, controlling for other covariates. In the final model, myometrial invasion at 1 cm was not independently associated with survival or relapse. Table 3 presents full details of the Cox regression analysis, including hazard ratios and confidence intervals.

**Table 3:** Cox proportional hazards regression analysis for overall survival

	Hazard ratio	P value	95% confidence interval	
			Lower	Upper
Age (years)	1.028	.019*	1.005	1.051
Invasion distance (1 Cm)	.725	.309	.389	1.348

^*^Indicates statistical significance at p < .05

To further explore why myometrial invasion was not found to be an independent prognostic factor in multivariate analysis, we performed a Chi-square comparison of key clinicopathological characteristics between patients with myometrial invasion ≥ 1 cm and those with < 1 cm (Table 4). Patients with deeper invasion (≥ 1 cm) were more likely to have high-grade tumors and lymphovascular space invasion (LVSI). Specifically, only 34.4% of patients with ≥ 1 cm invasion had FIGO grade 1 tumors, compared to 59.5% in the < 1 cm group (p = 0.002). Higher proportions of grade 2 (41.0% vs. 26.8%) and grade 3 (24.6% vs. 13.7%) tumors were observed among patients with ≥ 1 cm invasion. LVSI involvement was also significantly more frequent in the ≥ 1 cm group (45.9% vs. 12.2%, p < 0.001). No significant differences were found between the groups regarding age distribution (< 60 vs. ≥ 60 years, p = 0.520) or presence of severe comorbidities (p = 0.278).

**Table 4:** Chi-square analyisis: Comparison of study population Characteristics by Depth of Myometrial Invasion (< 1 cm vs. ≥ 1 cm)

Characteristic	< 1 cm invasion N = 205	≥ 1 cm invasion N = 61	P value
Age < 60 years	132 (64.4%)	42 (68.9%)	0.520
Severe comorbidity (Charlson’s comorbidity index > 4)	83 (40.5%)	20 (32.8%)	0.278
FIGO grade			
1	122 (59.5%)	21 (34.4%)	0.002
2	55 (26.8%)	25 (41.0%)	
3	28 (13.7%)	15 (24.6%)	
LVSI involvement	25 (12.2%)	28 (45.9%)	< 0.001

## Discussion

Endometrial cancer (EC) is one of the most common gynecological malignancies, and its incidence continues to rise globally. While the prognosis for early-stage EC is generally favorable, identifying reliable prognostic markers is crucial for tailoring treatment strategies and improving patient outcomes. Myometrial invasion (MI) is a critical prognostic factor influencing recurrence risk and overall survival.

In the current study, we evaluated the predictive value of the 50% cutoff for MI, a widely accepted threshold in the literature related to patient outcome, recurrence, and overall survival in early-stage EC. The FIGO staging system historically classified deep myometrial invasion using a one-third (33%) threshold before transitioning to a one-half (50%) cutoff in the 2009 update. Our results show that while 50% or more MI may relate to patient outcomes, it does not independently predict recurrence or overall survival. Interestingly, according to the results of our study, a cutoff of 33% MI provided stronger prognostication. Although the 50% cutoff has proven to be a reliable prognostic factor, our study suggests that the original 33% cutoff is a more accurate predictor of outcomes. This challenges the 2009 shift in the staging system and emphasizes the importance of further investigation.

In addition, we looked for an alternative for the current measurement method by evaluating several other MI parameters, such as absolute invasion depth and distance from uterine serosa. In this analysis, using absolute DOI with a cutoff of one centimeter appeared to correlate better with overall survival. DOI was our study’s most statistically significant parameter with the best correlation to outcome although it did not maintain statistical significance in multivariate analysis.

To better understand this finding, we conducted a Chi-square analysis comparing clinicopathological features by depth of invasion. This analysis revealed that deeper invasion was significantly associated with high-grade tumors and LVSI, offering an explanation to the lack of independent significance in the multivariate analysis.

Our findings agree with a study by Doghri et al. [20] which showed that DOI was superior to TFD as well as MI percentage as a prognostic factor. In their study the best DOI cutoff was found to be of 3 mm. Regarding distance from the serosa, our results are consistent with previously published results from a study by Oge et al. [18] that assessed 133 patients with early stage IB endometrioid endometrial cancer. The authors found that TFD did not significantly predict recurrence or survival outcomes and is not an independent prognostic factor in early EC patients. Contradicting results were presented by Chattopadhyay [17] demonstrating TFD is an independent predictor of survival and recurrence.

Our findings regarding alternative MI measurements are particularly relevant considering the 2023 FIGO staging revision, which takes under consideration the complexity of tumor assessment by incorporating multiple prognostic factors beyond the traditional 50% MI cutoff. Our finding that a 33% MI cutoff might be more prognostically significant than the traditional 50% is particularly interesting in the context of the evolving FIGO criteria.

The 2023 FIGO revision acknowledges that historical staging parameters, including the 50% MI cutoff, may need refinement as our understanding of endometrial cancer biology improves. This aligns with our observation that alternative measurements, such as absolute depth of invasion, might better predict outcome in early-stage disease. Furthermore, the new FIGO system’s emphasis on integrating multiple prognostic factors supports our approach of examining various invasion parameters rather than relying solely on percentage-based measurements.

### Study strengths and limitations

Our study benefited from relatively long follow-up as well as the review and merging of pathological and clinical data. This allowed us to capture important clinical outcomes and provide a detailed evaluation of prognostic factors in early-stage endometrial cancer (EC). It ensures that the correlations between myometrial invasion (MI) and patient outcomes are strong over time. Our sample size allowed for a thorough, detailed clinical and pathological analysis. The revision of pathological slides under question ensured the consistency and accuracy of our data.

However, the present study has some limitations as well. This is a single-center study, and the experience of other medical centers may be different. The Israeli population might have different features than the international patient population. Therefore, we acknowledge that more extensive multicenter studies would be beneficial in order to confirm our findings and detect additional prognostic factors. EC (especially early stage) is often an indolent disease, requiring large cohorts and extended follow-up period to fully capture the disease course and create more accurate prognostic models for early-stage endometrial cancer.

An additional limitation is the absence of comprehensive molecular data. The study period spans from 2006 to 2020, during which molecular profiling was not routinely available, especially in the earlier years. As a result, we were unable to incorporate molecular data into our analysis. We fully recognize the growing importance of integrating molecular classification into modern prognostic models, as reflected in recent guidelines. Future prospective studies with complete molecular data are needed to determine whether the prognostic impact of myometrial invasion differs across molecular subtypes.

## Conclusions

Our findings suggest that alternative cutoffs, particularly absolute myometrial invasion (MI) of 1 cm and optionally 33% MI cutoff, may offer better prognostic value for overall survival in early-stage endometrial endometrioid carcinoma compared to the traditionally used 50% cutoff. Furthermore, investigating various types of MI measures may contribute to ongoing efforts to refine prognostic markers in early-stage EC, potentially improving treatment strategies for future patients.

## Conflict of interest

The authors declare no competing interests.

## Ethical approval

This study was performed in line with the principles of the Declaration of Helsinki and approved by the Institutional Review Board of Soroka University Medical Center (Approval No. 0192-23-SOR).

## Data Availability

No datasets were generated or analysed during the current study.
